# Opposite regulatory effects of *Blautia massiliensis* and *Blautia faecis* on cognitive function, microglia and metabolite acetic acid in mice

**DOI:** 10.3389/fneur.2026.1854107

**Published:** 2026-06-24

**Authors:** Minghui Li, Na Wu, Weidong Yu, Xin Wang, Yang Zhao, Xuyang Ge, Zhuran Zhao, Xinjuan Wang, Jingzhu Guo

**Affiliations:** 1 Department of Pediatric, Peking University People’s Hospital, Beijing, China; 2 Department of Central Laboratory & Institute of Clinical Molecular Biology, Peking University People’s Hospital, Beijing, China

**Keywords:** acetate, *Blautia*, cognitive function, gut microbiota, neuroinflammation

## Abstract

Cognitive impairment is a significant health problem worldwide, closely associated with the status of gut microbiota. Our recent research has revealed the *Blautia faecis* and *Blautia massiliensis* exhibit opposing associations with cognitive function in children with Down syndrome clinically characterized by cognitive dysfunction. However, the role and mechanisms of *Blautia faecis* and *Blautia massiliensis* in cognitive function remain unknown. Therefore, we gavaged C57BL/6 male mice with commercially available *Blautia faecis* and *Blautia massiliensis* for 3 weeks and assessed cognitive function using the novel object recognition and Y-maze test. *Blautia faecis* administration impaired cognitive performance, whereas *Blautia massiliensis* treatment improved it, with these effects observed predominantly in the absence of antibiotic pretreatment. Furthermore, we observed that administration of *Blautia faecis* increased the number of microglia, resulting in a twofold increase in cell count relative to WT control. Hippocampal pro-inflammatory cytokines were significantly upregulated in the *Blautia faecis* group, while *Blautia massiliensis* suppressed these neuroinflammatory responses. Notably, *Blautia massiliensis* produces approximately six times more acetate than *Blautia faecis*. Consequently, our findings indicate that *Blautia massiliensis* enhances cognitive function whereas *Blautia faecis* impairs it, and we speculate that differential acetate production may contribute to these opposing effects.

## 1 Introduction

Cognitive impairment, a prevalent and progressive neurocognitive syndrome marked by declining memory, attention, and executive functions, represents a major global public health challenge with profound impacts on individual quality of life and healthcare systems (1). The “Gut-brain” axis has been reported to solve the function barrier related to the brain. Studies have shown that probiotic supplementation can improve cognitive impairment in patients with Alzheimer’s disease (2, 3). It suggests bacteria play a key role in the development of neurodevelopmental disorders (4, 5).

In our earlier study, we assessed the cognitive levels of Chinese children with Down syndrome using the Full Scale IQ test and collected the fecal samples for 16S rRNA sequencing. Cognitive impairment is one of the most central and characteristic clinical manifestations in children with Down syndrome. The results revealed significant differences in gut microbiota composition between Down syndrome children and healthy controls (6). Specifically, the abundance of *Blautia massiliensis* showed a positive correlation with the children’s cognitive levels, while the abundance of *Blautia faecis* showed a negative correlation (7). *Blautia massiliensis*, an abundant resident of the healthy human gut microbiome, is distinguished by its extensive saccharolytic capacity, capable of fermenting a diverse array of complex carbohydrates. This robust metabolic profile highly supports a superior potential for SCFA production. In contrast, *Blautia faecis* exhibits a substantially more restricted carbohydrate fermentation profile, suggesting different metabolic constraints and ecological roles within the intestinal environment (8, 9). The original taxonomic descriptions highlight profound differences in their metabolic profiles and ecological niches for *Blautia massiliensis* and *Blautia faecis*.

Recent functional metagenomic analyses further underscore that alterations in the *Lachnospiraceae* family and their metabolic pathways are closely associated with various neurodegenerative disorders (10). *Blautia* degrades polysaccharides into short-chain fatty acids (SCFAs) that play a vital role in the development and homeostasis of the central nervous system (8, 9, 11, 12). Among these SCFAs, acetate is particularly noteworthy for its multifaceted neuroactive properties. Upon crossing the blood–brain barrier, acetate serves as a crucial energy substrate for astrocytes, acts as a potent modulator to suppress microglial activation, and functions as an epigenetic regulator via the inhibition of histone deacetylases (HDACs) (13–15). Moreover, SCFAs produced from *Blautia* could suppress neuroinflammation and attenuate neurocognitive disorders (16). This highlights the potential of *Blautia* in mitigating cognitive impairment, although the precise mechanisms remain to be fully elucidated for *Blautia massiliensis* and *Blautia faecis* from the species level. Based on our previous clinical correlations, we hypothesize that *Blautia massiliensis* and *Blautia faecis* exert distinct regulatory effects on cognitive function through SCFAs mediated pathways.

Therefore, healthy adult male mice were utilized to evaluate the effects of these two strains on cognitive function and the underlying mechanisms. By employing interventions in healthy wild-type mice, we aimed to eliminate the complex genetic confounding associated with Down syndrome, thereby isolating the direct neurocognitive effects attributable to the strains themselves. Although the intact cognitive baseline in these mice may present a ceiling effect, this proof-of-concept model remains an indispensable initial step in our research. We administered *Blautia massiliensis* and *Blautia faecis* to C57BL/6 mice via oral gavage for 3 weeks. We then evaluated cognitive function using the novel object recognition and Y-maze tests, and analyzed both the metabolic byproducts of the bacteria and the activation status of hippocampal microglia.

## 2 Materials and methods

### 2.1 Preparation of *Blautia faecis* and *Blautia massiliensis*

*Blautia faecis* (DSM 27629) and *Blautia massiliensis* (DSM 101187), purchased from Mingzhou Bio Ltd. (Ningbo, China), were cultivated anaerobically at 37 °C in DSMZ Medium 104 (Mingzhou Bio Ltd., KDM144). Following the glycerol stock activation, the overnight culture was diluted with fresh medium to a standardized optical density (OD_600_ = 1.0). A subculture was then prepared by inoculating 100 μL of this standardized suspension into a new 10 mL of fresh medium, which was incubated anaerobically for 10 h to reach the late logarithmic growth phase. The bacterial cells were then harvested by centrifugation at 4000 × g for 10 min at 4 °C. The supernatant was discarded, and the bacterial pellet was washed once with sterile, anaerobic phosphate-buffered saline (PBS, pH 7.4) and finally resuspended in the same buffer to a final concentration of 1 × 10^9^ CFU/mL for subsequent *in vivo* administration.

### 2.2 Animals

Male C57BL/6J mice (12 weeks of age) were provided by Viewsolid Biotech Ltd. In this proof-of-concept study, we exclusively used male mice to avoid the confounding effects of the female estrous cycle. Because hormonal fluctuations can introduce substantial variance in behavioral and neuroinflammatory outcomes, this design provided a standardized baseline to isolate the intrinsic effects of the *Blautia* interventions (17, 18). Mice were housed in groups of 6 per cage and maintained in an animal facility under conditions of 22 ± 2 °C, 55 ± 10% humidity, and a 12-h light/dark cycle. Standard rodent chow and water were available ad libitum. Every effort was made to reduce the number of animals used and minimize animal suffering. In strict adherence to institutional animal ethics and the 3Rs principles (Replacement, Reduction, and Refinement) for exploratory *in vivo* studies, the sample size was constrained to minimize animal usage. An *a priori* power analysis for our 2 × 2 factorial design using G*Power software (version 3.1.9.7) indicated that a total sample size of 128 mice (*n* = 32 per group) would be required to detect a conventional medium effect size (*f* = 0.25) with 80% power at an alpha level of 0.05. Given these constraints, a balanced cohort of n = 6 mice per group was established to investigate initial, high-magnitude biological responses. We acknowledge that this ethically reduced cohort limits the statistical power to capture subtle or medium-sized variations, and non-significant trends are evaluated conservatively. Animals were weighed regularly to ensure they maintained a stable body weight. In addition, they were visually inspected periodically for signs of disease or injury. Throughout the experimental period, no unexpected adverse events or mortalities occurred, and no animals were excluded from the final statistical analysis. All animal studies and experimental procedures were approved by the Ethical Review Committee of Peking University People’ Hospital. All animal experiments were reported in accordance with the ARRIVE 2.0 guidelines (Supplementary File S1).

After habituation and weight-matching to ensure equal baseline body weights across conditions, mice were randomly assigned to the six experimental groups across two independent batches (for *Blautia faecis* and *Blautia massiliensis*, respectively). The randomisation sequence was generated using a computer-based random number generator (GraphPad Prism 8.0.1), which allocated the weight-matched animals into their respective treatment blocks: (1) WT (sterile PBS without ABX, *n* = 6/batch, total *n* = 12); (2) *Blautia faecis* (without ABX, *n* = 6); (3) *Blautia massiliensis* (without ABX, *n* = 6); (4) ABX (sterile PBS with ABX, *n* = 6/batch, total *n* = 12); (5) ABX + *Blautia faecis* (*n* = 6); and (6) ABX + *Blautia massiliensis* (*n* = 6). Control data were not pooled for analysis; each bacterial intervention was statistically compared exclusively to its parallel control group (*n* = 6). To establish the commensal bacteria-depleted model, ABX-treated mice received a daily oral gavage of an antibiotic cocktail (1 mg/mL ampicillin, 1 mg/mL neomycin, 0.5 mg/mL vancomycin, and 1 mg/mL metronidazole) for 7 consecutive days prior to bacterial interventions.

**Figure 1:**
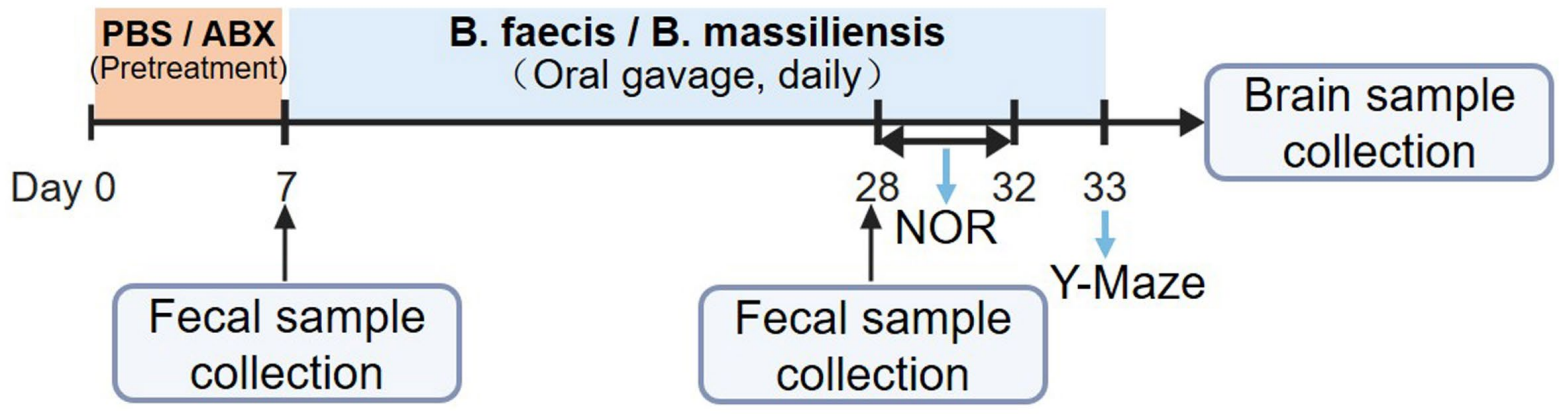
Flow chart of animal experiments.

Microbiota transplantation was initiated on day 8. Mice in the bacterial intervention groups received 1.0 × 10^9^ colony-forming units (CFU) of either *Blautia faecis* or *Blautia massiliensis* suspended in 200 μL of sterile PBS via oral gavage once daily for 3 weeks. Control animals received 200 μL of sterile PBS alone. Behavior tests were performed after 3 weeks of microbiota transplantation. The daily treatments were maintained continuously throughout the entire behavioral testing period (19). The schematic experimental design for this study is shown in Figure 1.

### 2.3 Behavioral tests

Following the initial 3-week bacterial intervention period and the second fecal sample collection on Day 28, the Novel Object Recognition (NOR) test (Days 28–32) and the Y-maze test (Day 33) were conducted to evaluate learning and memory functions. To prevent carryover effects and minimize cumulative stress, an interval of at least 24 h was implemented between different behavioral tests. On testing days, mice were transferred to the testing room and allowed to habituate in their home cages for 1 h prior to the experiments to reduce environmental stress. The ambient light intensity in the testing room was controlled at 60 lux unless otherwise specified. To eliminate residual olfactory cues, all experimental arenas and apparatuses were thoroughly cleaned with 75% ethanol between individual trials. To ensure strict objectivity, all behavioral testing procedures and subsequent data analyses were conducted by investigators completely blinded to the experimental group assignments.

#### 2.3.1 Novel object recognition test

The Novel Object Recognition (NOR) test was conducted to assess short-term object recognition memory (20) in a rectangular open-top arena (40 cm × 40 cm × 30 cm). The testing protocol consisted of three distinct phases: habituation, familiarization (acquisition), and retention (21). During the habituation phase, which spanned three consecutive days, each mouse was placed facing the wall at the edge of the empty arena and allowed to freely explore for 10 min daily. During the familiarization phase, two identical, odorless objects were firmly secured 10 cm from the side walls. Mice were introduced into the arena facing away from the objects at an equidistant point and allowed to explore for 5 min. Following an inter-trial interval of 30 min, the retention phase was conducted. One of the familiar objects was replaced with a novel object of a different shape, and the mice explored the arena for another 5 min. Animal movements and object exploration times were automatically tracked and recorded using SMART v3.0 behavioral analysis software. Active object exploration was defined as the mouse’s nose being directed toward the object within a distance of ≤ 2 cm. Cognitive performance was evaluated by calculating the Discrimination Index (DI): DI = (Time exploring novel object − Time exploring familiar object)/(Time exploring novel object + Time exploring familiar object).

#### 2.3.2 Y-maze

The Y-maze test was conducted to evaluate spatial working memory, specifically spontaneous alternation behavior. Prior to testing, the maze was thoroughly cleaned to ensure it was strictly odorless and free of any residual excretions. For each trial, the mouse was gently handled, facing away from the experimenter, and carefully placed at the distal end of the designated start arm (Arm A), facing the wall. The experimenter immediately exited the testing area to avoid human interference. The mouse was then allowed to freely explore the three arms of the maze for a continuous duration of 5 min. Animal movements and the sequence of arm entries were automatically tracked and recorded using SMART v3.0 behavioral analysis software. A valid arm entry was strictly defined as all four paws of the mouse fully entering the designated arm. Upon completion of the trial, the animal was returned to its home cage, and the apparatus was meticulously sanitized with 75% ethanol and dried with paper towels to eliminate olfactory cues for the next subject. The initial placement arm (Arm A) was excluded from the entry sequence. To quantify spatial working memory, the percentage of spontaneous alternation was calculated using the standard formula: Spontaneous Alternation (%) = [Number of actual alternations/(Total number of arm entries − 2)] × 100.

### 2.4 Genomic DNA extraction, gel purification and sequencing for *Blautia faecis and Blautia massiliensis*

To evaluate the intestinal colonization of *Blautia faecis* and *Blautia massiliensis*, fresh fecal samples were collected from the experimental animals on Day 7 (post-antibiotic pretreatment, prior to bacterial gavage) and Day 28 (following 3 weeks of intervention and prior to behavioral testing). Total genomic DNA was extracted from the feces using a Fecal Genomic DNA Extraction Kit (Cat. No. DP328; TIANGEN Biotech, Beijing, China). Genomic DNA extracted from the cultured bacterial pellets using a Bacterial DNA Extraction Kit (Cat. No. DP302; TIANGEN Biotech, Beijing, China), served as the control. PCR amplification was conducted using species-specific primers of *Blautia faecis* (Forward: CGGATTCCCTA CGAAGAGGC; Reverse: CAAGCGGTGTGACCAAAAGG) and *Blautia massiliensis* (Forward: AAAACCGGATGGACCGTTGT; Reverse: GCTCCGTCAAAATGCCCTTC). The amplification was performed in a 25 μL reaction volume consisting of 12.5 μL of 2 × PrimeSTAR® Max DNA Polymerase (Code No. R045A; Takara Bio, Kusatsu, Japan), 1.0 μL of each forward and reverse primer (10 μM), 1.0 μL of genomic DNA template, and 9.5 μL of sterile double-distilled water (ddH₂O). The PCR cycling conditions included an initial denaturation at 98 °C for 10 min, followed by 30 cycles of denaturation at 98 °C for 15 s, annealing at 58 °C for 30s, and extension at 72 °C for 90 s, with a final extension at 72 °C for 10 min. The amplified PCR products were mixed with 6 × DNA Loading Buffer (Cat. No. RT201-01; TIANGEN Biotech) and subjected to electrophoresis on a 1% (w/v) agarose gel. Following electrophoresis, the positive controls (*Blautia faecis* and *Blautia massiliensis*) were excised from the gel, purified, and subsequently sequenced using an ABI 3730XL Genetic Analyzer (Applied Biosystems, Foster City, CA, USA) to verify the sequence integrity and species identity.

### 2.5 Immunofluorescence

Following the completion of all behavioral tests on Day 34, the experimental cohorts (n = 6 mice per group) were randomly divided into two equal sub-cohorts to accommodate mutually exclusive tissue processing requirements. Specifically, 3 mice per group were deeply anesthetized with ketamine (100 mg/kg) and xylazine hydrochloride (10 mg/kg) and perfused intracardially with 4% paraformaldehyde (PFA) in 0.1 M phosphate buffer (pH 7.4) for immunofluorescence analysis. The brains were removed, postfixed with 4% PFA for 12 h at 4 °C, and then dehydrated consecutively in 20 and 30% sucrose solutions. Coronal sections (30 μm) were sliced using a cryostat microtome (model 1950, Leica). Free-floating sections were washed in phosphate-buffered saline (PBS) and blocked with buffer that contained 5% normal goat serum and 0.3% Triton X-100 for 1 h at room temperature (22). Following this blocking step, the sections were incubated overnight at 4 °C without agitation with a recombinant rabbit monoclonal anti-Iba1 primary antibody (Clone EPR16588; Abcam, Cambridge, UK) diluted in 1% goat serum.

On the following day, the sections were washed three times for 10 min each in PBST. They were then incubated with a Cy3-conjugated goat anti-rabbit IgG secondary antibody (Cat. No. GB21303; Servicebio, Wuhan, China) diluted in 1% goat serum for 1.5 h at room temperature in the dark. After three additional 10-min washes with PBST, cell nuclei were counterstained using DAPI (Cat. No. D9542; Sigma-Aldrich, St. Louis, MO, USA). Finally, the sections were cover-slipped using a mounting medium (stored at −20 °C). Images were taken using a laser-scanning confocal microscope (LSM800, Zeiss, Germany).

For the quantitative analysis of microglia, the acquired immunofluorescence images were analyzed using ImageJ software (National Institutes of Health, Bethesda, MD, USA). Briefly, the hippocampal CA1 region was anatomically defined as the region of interest (ROI) with the assistance of DAPI counterstaining. The Iba1-positive cells within the CA1 region were then manually counted using the Multi-point tool. For each animal, at least three representative non-adjacent coronal sections containing the hippocampus were analyzed.

### 2.6 Quantitative reverse transcription PCR (RT-qPCR)

The brains for remaining three mice per group were rapidly removed and placed on an ice-cold surgical plate after deeply anesthetized and sacrificed on Day 34. The bilateral hippocampus was meticulously microdissected, immediately snap-frozen in liquid nitrogen, and subsequently stored at −80 °C until further processing for RNA extraction.

To evaluate the mRNA expression levels of microglia-related markers and inflammatory cytokines, total RNA was extracted from the hippocampal tissues using the TaKaRa MiniBEST Universal RNA Extraction Kit (Cat. No. 9767; Takara Bio, Kusatsu, Japan) according to the manufacturer’s instructions. To eliminate potential genomic DNA contamination and synthesize first-strand cDNA, the Thermo Scientific Revert Aid Reverse Transcriptase Kit with DNase I (Cat. No. K16225; Thermo Fisher Scientific, Waltham, MA, USA) was utilized. Quantitative real-time PCR was subsequently performed on an ABI 7500 Fast Real-Time PCR System (Applied Biosystems, Foster City, CA, USA) using the SYBR Green Realtime PCR Master Mix (Cat. No. QPK-201; TOYOBO, Osaka, Japan). The specific primer sequences used for amplification were as follows: *Iba-1* (Forward: 5’-TCTGCCGTCCAAACTTGAAGCC-3′; Reverse: 5’-CTCTTCAG CTCTAGGTGGGTCT-3′), *Tmem119* (Forward: 5’-CCTTCACCCAG AGCTGGTTC-3′; Reverse: 5’-GGCTACATCCTCCAGGAAGG-3′), *IL-1β* (Forward: 5’-AAGGCAGTGGAGCAGGTGAA-3′; Reverse: 5’-CCAGCAGACTCAATACACAC-3′), and the internal reference gene GAPDH. To minimize technical variations during cDNA synthesis, the input concentration of total RNA was standardized across all samples. All PCR reactions were performed in triplicate. The relative mRNA expression levels of the target genes were calculated using the 2^^−(∆∆Ct)^ method and normalized to *GAPDH*. *GAPDH* was selected as the internal reference gene because it is a universally established housekeeping gene in neurobiology, and current literature within the gut-brain axis field widely accepts its biological stability under microbiota interventions. For relative quantification, the corresponding vehicle control group within each experimental block (the WT group for standard cohorts and the ABX group for antibiotic-pretreated cohorts) was designated as the calibrator with a reference value of 1.0.

### 2.7 Targeted metabolomic analysis of short-chain fatty acids (SCFAs)

To evaluate the production of short-chain fatty acids (SCFAs) by *Blautia faecis* and *Blautia massiliensis*, targeted metabolomic analysis was performed on the bacterial culture supernatants. An aliquot of the supernatant was placed into a 1.5 mL tube and acidified with 0.05 mL of 50% H_2_SO_4_. Subsequently, 0.2 mL of an extraction solution (methyl tert-butyl ether) containing 25 mg/L of 2-methylvaleric acid as an internal standard was added. The mixture was vortexed for 30 s, oscillated for 10 min, and subjected to ultrasonication in an ice-water bath for 10 min. Following incubation at −20 °C for 30 min, the samples were centrifuged at 10,000 rpm for 15 min at 4 °C.

The resulting supernatant was transferred to a 2 mL glass vial for quantification using an Agilent 7890B-5977B Gas Chromatography–Mass Spectrometry (GC–MS) system (Agilent Technologies, Santa Clara, CA, USA) equipped with an HP-FFAP capillary column (30 m × 250 μm × 0.25 μm). Helium was utilized as the carrier gas at a constant flow rate of 1.2 mL/min. A 1 μL aliquot was injected in split mode with a split ratio of 5:1. The oven temperature program was initialized at 50 °C for 1 min, ramped to 150 °C at a rate of 50 °C/min for 1 min, and further incrementally raised to a final temperature of 240 °C. The injection, transfer line, quadrupole, and ion source temperatures were maintained at 220 °C, 240 °C, 150 °C, and 240 °C, respectively. The concentrations of 11 targeted SCFAs (including acetic, propionic, butyric, isobutyric, valeric, isovaleric, hexanoic, heptanoic, octanoic, nonanoic, and decanoic acids) were calculated and quantified based on standard curves generated from commercially available reference standards.

### 2.8 Statistical analysis

All data were analyzed using GraphPad Prism 8.0.1 software. The data were presented as the mean ± SEM. Unpaired two-tailed *t* test were used (as illustrated in figure legends). Values of *p* < 0.05 were considered statistically significant.

## 3 Results

### 3.1 Successful colonization and verification of *Blautia faecis* and *Blautia massiliensis*

**Figure 2:**
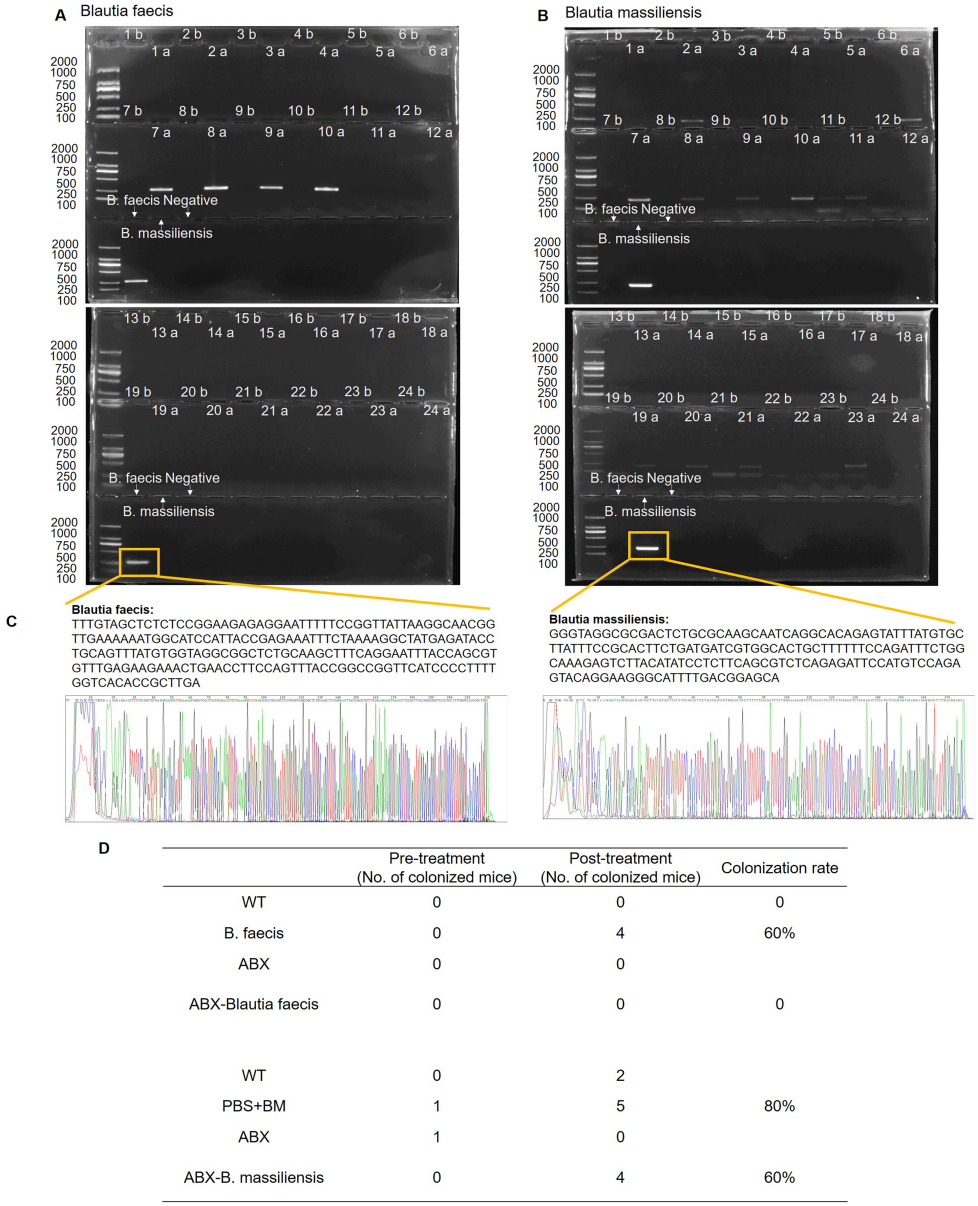
Intestinal colonization of *Blautia faecis* and *Blautia massiliensis* and validation of strain identity. **(A,B)** PCR-based detection of intestinal colonization for **(A)** *Blautia faecis* and **(B)** *Blautia massiliensis* in fecal samples collected before (labeled as ‘b’) and after (labeled as ‘a’) the 3-week intervention period, where lanes 1–6 represent Vehicle (PBS), 7–12 represent PBS + *Blautia*, 13–18 represent ABX, and 19–24 represent ABX + *Blautia* groups. **(C)** Molecular identification of the administered strains showing representative Sanger sequencing chromatograms and corresponding nucleotide sequences for *B. faecis* (left) and *B. massiliensis* (right) amplified using species-specific primers. The positive controls (*B. faecis* and *B. massiliensis*) are PCR products amplified from genomic DNA extracted from their respective pure cultures. The negative control is the amplification product using nuclease-free water as the template. **(D)** Table of colonization success rates of *Blautia faecis* and *Blautia massiliensis.*

To assess whether the orally administered *Blautia* strains could survive and successfully engraft in the murine gastrointestinal tract, we extracted fecal genomic DNA before and after the 3-week intervention period and performed species-specific PCR analysis. As shown in Figures 2A,B, prior to the intervention (labeled as ‘b’), the target *Blautia* sequences were largely absent across the experimental groups. After the continuous oral gavage (labeled as ‘a’), PCR analysis confirmed the presence of *Blautia faecis* (Figure 2A) and *Blautia massiliensis* (Figure 2B) in the treated mice. Notably, trace *Blautia massiliensis* signals were also detected in one pre-treatment animal and two post-treatment WT controls (Figure 2D). Because SPF mice are not germ-free, these signals are biologically normal and reflect the natural background carriage of this commensal microbe in the colony.

Genomic DNA was extracted from the procured bacterial strains as an internal control. To confirm the accurate identity and purity of the administered *Blautia* strains, Sanger sequencing analysis of the amplified products yielded high-quality chromatograms with clear, distinct peaks, indicating the high purity of the amplicons (Figure 2C). The obtained nucleotide sequences perfectly matched the specific target regions of *Blautia faecis* and *Blautia massiliensis*, respectively. Statistical analysis revealed that the colonization efficiency in the absence of antibiotic pretreatment was substantially greater than that in the ABX-pretreated group, with the former exceeding 60% (Figure 2D).

These molecular detection results verify that the administered *Blautia faecis* and *Blautia massiliensis* strains successfully colonized the gut, validating the efficacy of our *in vivo* intervention model.

### 3.2 *Blautia massiliensis* enhances cognitive function, whereas *Blautia faecis* impairs it in mice

**Figure 3:**
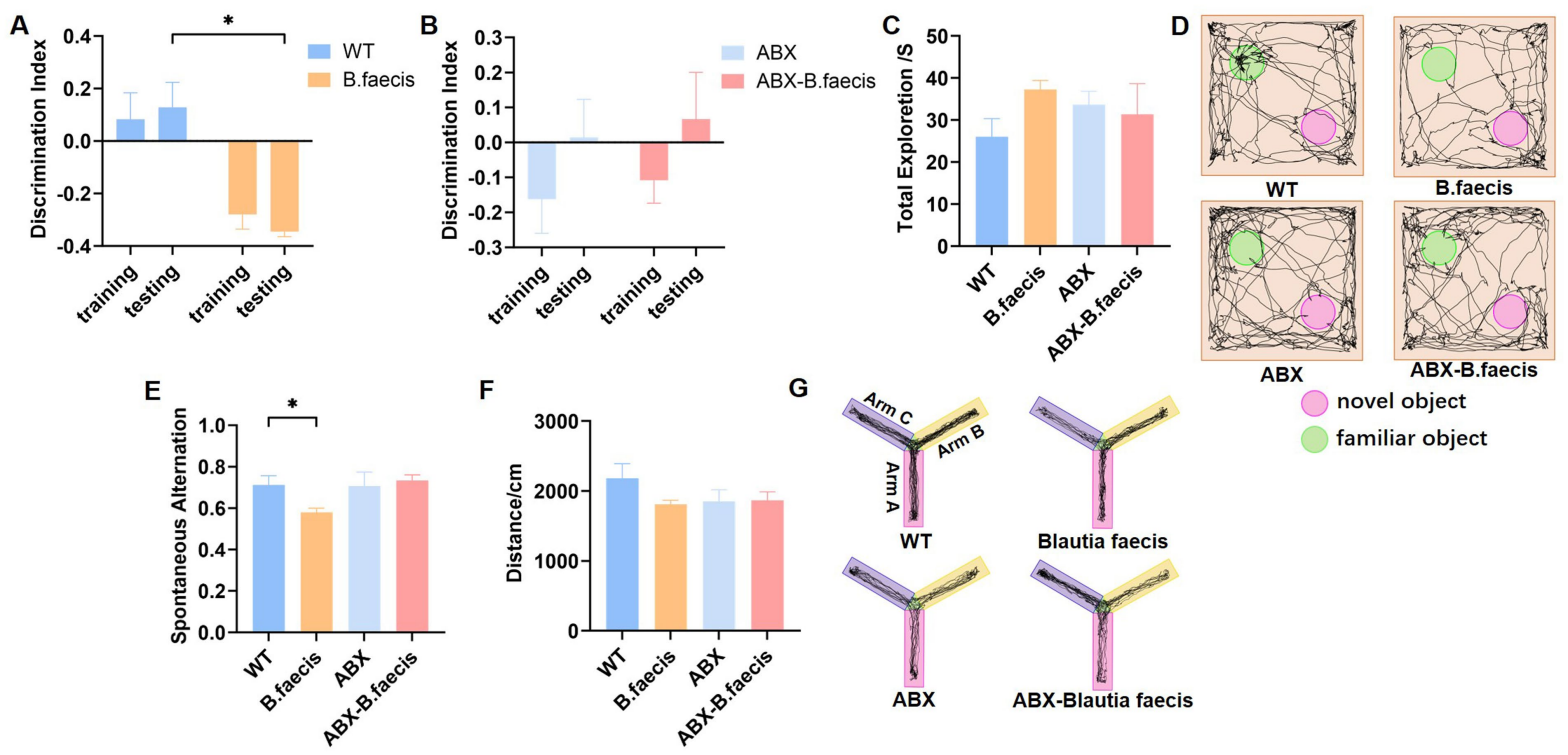
*Blautia faecis* impairs cognitive function in mice. **(A–D)** NOR test for *B. faecis* including **(A)** discrimination index of mice without antibiotic pretreatment and **(B)** mice with ABX pretreatment during training and testing phases. Two-way ANOVA with Bonferroni’s *post hoc* test for **(A,B)**, *p < 0.05 compared to WT group. **(C)** Total exploration time and **(D)** representative movement tracking maps in the NOR test. **(D)** The green circle indicates the familiar object, and the pink circle indicates the novel object (replaced object). **(E–G)** Y-maze test for *B. faecis* showing **(E)** spontaneous alternation percentage, **(F)** total distance traveled and **(G)** tracking maps. Unpaired Student’s *t* test for **(C,E,F)**, *p < 0.05 compared to WT group. **(G)** The three arms of the Y-maze are labeled as Arm A, Arm B, and Arm C. All data are presented as Mean ± SEM (*n* = 6 mice per group).

To evaluate the effects of *Blautia faecis* and *Blautia massiliensis* on cognitive function, novel object recognition and Y-maze test were conducted. Mice without antibiotic (ABX) treatment exhibited cognitive impairment in both novel object recognition test and Y-maze test after *Blautia faecis* gavage, which was manifested by a decrease in Discrimination Index (Session: *F_1,5_* = 0.05329, *p* = 0.8266, $\eta_{p}^{2}$ = 0.001; Group: *F_1,5_* = 29.06, *p* = 0.0030, $\eta_{p}^{2}$ = 0.605; Session × Group: *F_1,5_* = 0.4604, *p* = 0.5276, $\eta_{p}^{2}$ = 0.026; Figures 3A,D) and a reduction in Spontaneous Alternation (*t_10_* = 2.721, *p* = 0.0215, Cohen’s d = 1.5712; Figures 3E,G). In contrast, mice pretreated with ABX prior to *Blautia faecis* gavage exhibited no statistically significant changes in either the Discrimination Index (Session: *F_1,5_* = 1.888, *p* = 0.2278; Group: *F_1,5_* = 0.2972, *p* = 0.6091; Session × Group: *F_1,5_* = 4.966e-005, *p* = 0.9946; Figures 3B,D) and Spontaneous Alternation (Figures 3E,G). ABX pretreatment had no significant impact on the Discrimination Index and Spontaneous Alternation (Figures 3A,B,E).

**Figure 4:**
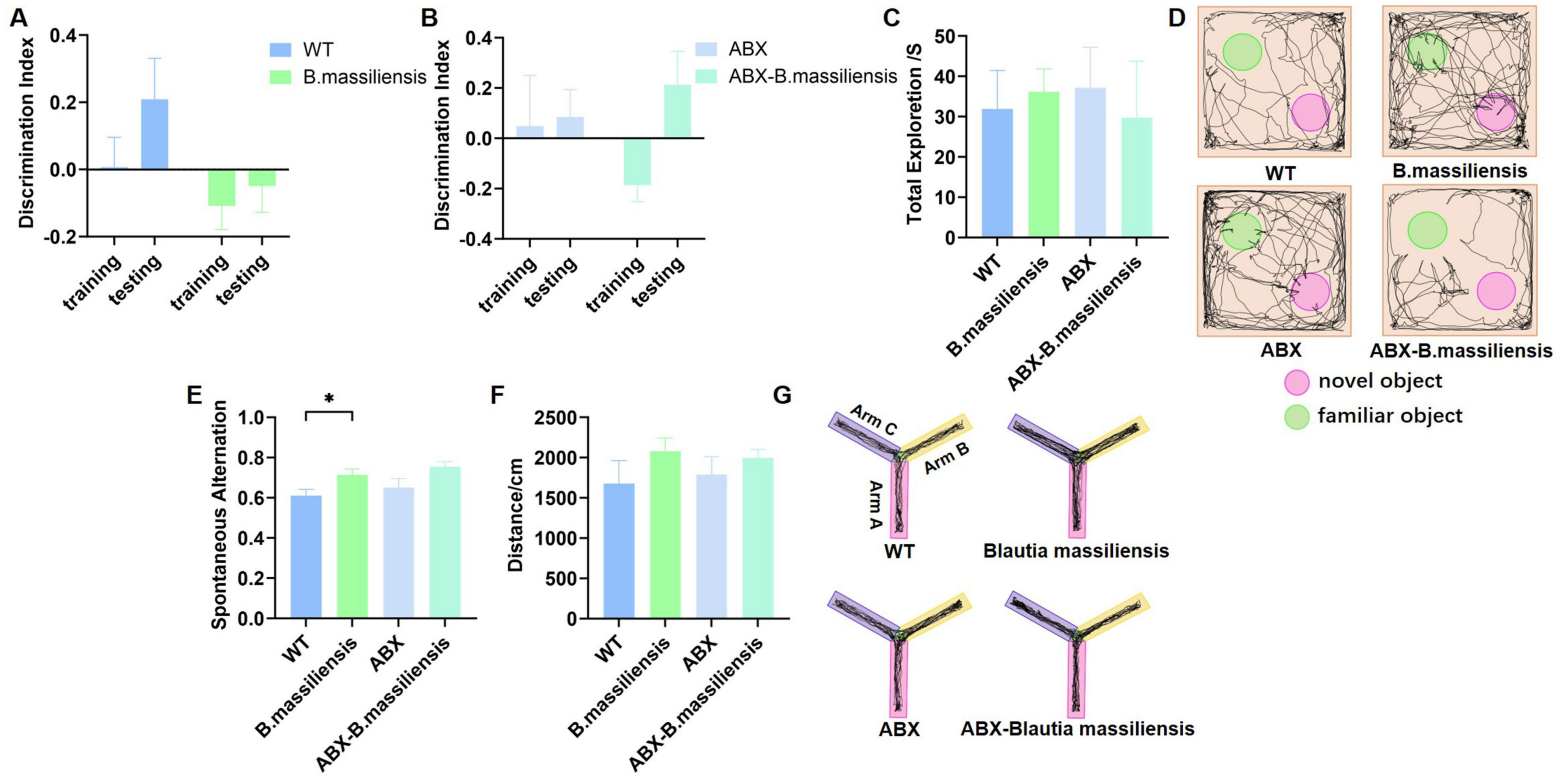
*Blautia massiliensis* ameliorates cognitive function in mice. **(A–D)** NOR assessments for *B. massiliensis* including **(A)** discrimination index of mice without antibiotic pretreatment, **(B)** mice with ABX pretreatment during training and testing phases. Two-way ANOVA with Bonferroni’s post hoc test for **(A,B)**. **(C)** Total exploration time and **(D)** representative tracking maps. **(D)** The green circle indicates the familiar object, and the pink circle indicates the novel object (replaced object). **(E–G)** Y-maze assessments for *B. massiliensis* showing **(E)** spontaneous alternation percentage, **(F)** total distance traveled, and **(G)** tracking maps. Unpaired Student’s t test for **(C,E,F)**, *p < 0.05 compared to WT group. **(G)** The three arms of the Y-maze are labeled as Arm A, Arm B, and Arm C. All data are presented as Mean ± SEM (*n* = 6 mice per group).

In the *Blautia massiliensis* gavage group, spontaneous alternation was markedly increased in the Y-maze test in the non-ABX subgroup (*t_10_* = 2.387, *p* = 0.0382, Cohen’s d = −1.3780; Figures 4E,G). Regarding the discrimination index in the novel object recognition test, Two-way ANOVA revealed a significant main effect of the treatment; however, subsequent Bonferroni post-hoc comparisons failed to detect significant intergroup differences (Session: *F_1,5_* = 2.080, *p* = 0.2088, $\eta_{p}^{2}$ = 0.092; Group: *F_1,5_* = 49.52, *p* = 0.0009, $\eta_{p}^{2}$ = 0.171; Session × Group: *F_1,5_* = 0.4439, *p* = 0.5347, $\eta_{p}^{2}$ = 0.029; Figures 4A,D). This discrepancy may be attributed to the relatively small sample size. These findings suggest that *Blautia massiliensis* may exert a beneficial effect on cognitive function improvement. Conversely, the ABX-pretreated group showed no significant differences in either behavioral paradigm compared to controls (Session: *F_1,5_* = 4.704, *p* = 0.0823; Group: *F_1,5_* = 0.1851, *p* = 0.6849; Session × Group: *F_1,5_* = 1.954, *p* = 0.2211; Figures 4B,D,E,G), which may be attributable to the relatively small sample size (*n* = 6). Similarly, ABX pretreatment in the *Blautia massiliensis* gavage group had no significant impact on the Discrimination Index and Spontaneous Alternation (Figures 4A,B,E). Furthermore, no statistically significant differences were observed in the motorability of mice across any experimental groups (Figures 3C,F, 4C,F), while this suggests that gross locomotive function remained largely unaffected by the interventions.

### 3.3 *Blautia massiliensis* reduces microglia numbers and suppresses inflammation, while *Blautia faecis* increases them

Given the distinct behavioral outcomes induced by the two *Blautia* strains, we next investigated their regulatory effects on neuroinflammation, specifically focusing on microglial activation in the hippocampus. To accurately characterize microglial functional states, we evaluated two complementary markers: Iba-1 and Tmem119. Iba-1 is a classic pan-microglial marker that is markedly upregulated during neuroinflammatory activation (23). In contrast, Tmem119 is a highly specific protein primarily restricted to brain-resident, homeostatic microglia (24). Co-evaluating these proteins allows us to robustly quantify the total microglial pool and distinguish endogenous neuroimmune shifts from a complete loss of homeostasis.

**Figure 5:**
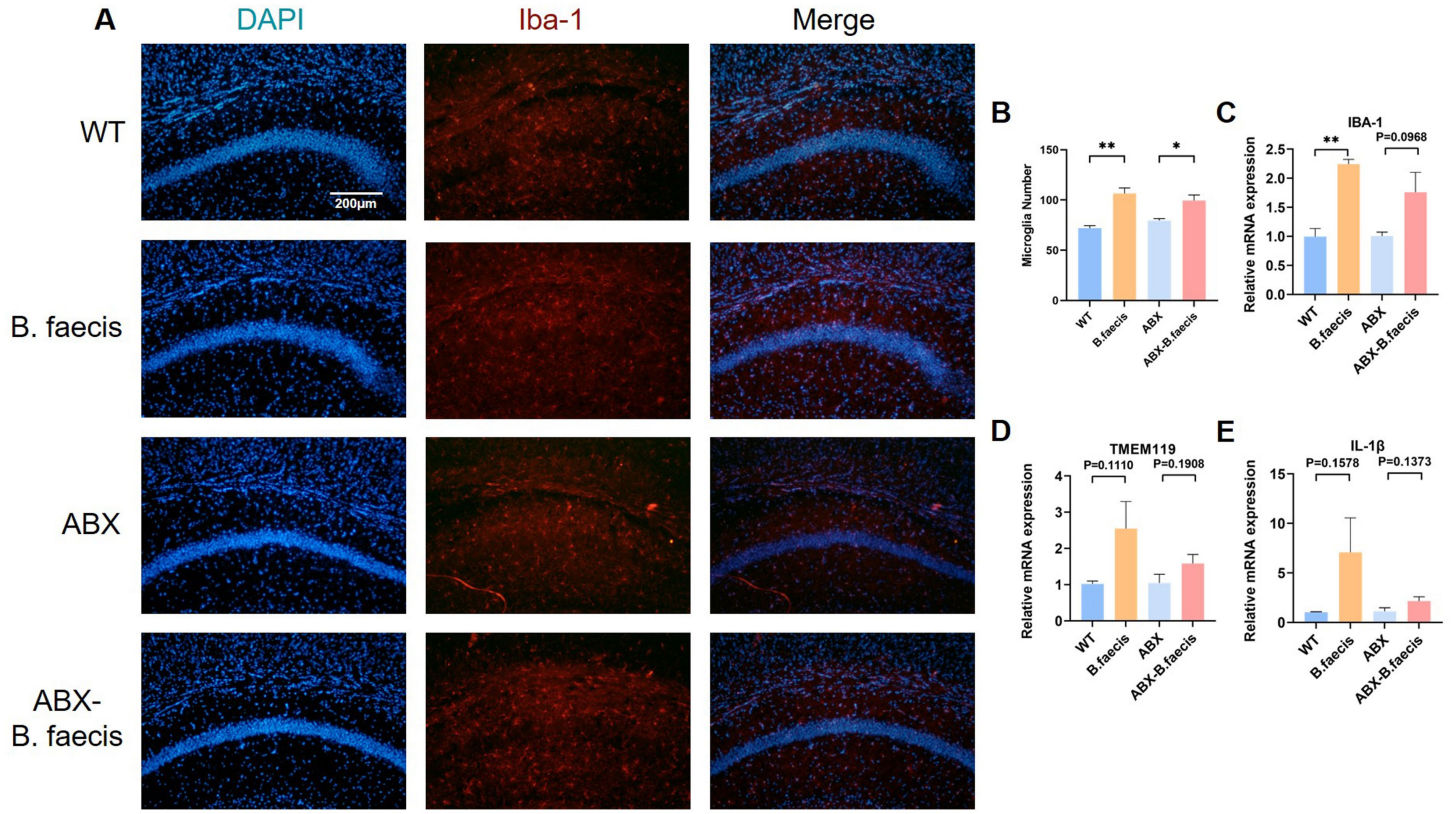
*Blautia faecis* induces an increase in microglia and pro-inflammatory factors in the hippocampus. **(A)** Representative immunofluorescence images of DAPI (blue) and Iba-1 (red) staining in the hippocampal regions of WT and ABX mice treated with or without *B. faecis*, Scale bar, 200 μm. WT: wild-type mice pretreated with PBS. **(B)** Quantitative analysis of Iba-1 positive microglia numbers and relative mRNA expression levels of *Iba-1* **(C)**, *Tmem119* **(D)**, and *IL-1β* **(E)** as determined by qPCR normalized to *GAPDH,* with the corresponding vehicle control group set as the calibrator (relative value = 1.0). Specific *p* values for mRNA expression include: *Iba-1* (*p* < 0.01 for *B. faecis* vs. WT; *p* = 0.0968 for ABX-*B. faecis* vs. ABX), *Tmem119* (*p* = 0.1110 for *B. faecis* vs. WT; *p* = 0.1908 for ABX-*B. faecis* vs. ABX), and *IL-1β* (*p* = 0.1578 for *B. faecis* vs. WT; *p* = 0.1373 for ABX-*B. faecis* vs. ABX). Data are expressed as Mean ± SEM (*n* = 3 per group), and statistical significance was analyzed via Student’s *t*-test, *p < 0.05 and **p < 0.01.

As shown in Figures 5A,B (*t_4_* = 5.850, *p* = 0.0043, Cohen’s d = −4.7769), administration of *Blautia faecis* to mice without ABX treatment significantly increased the number of Iba-1 positive microglia indicative of a pro-inflammatory activated state. This microglial proliferation was also significantly exacerbated by *Blautia faecis* in the ABX-pretreated model (*t_4_* = 3.523, *p* = 0.0244, Cohen’s d = −2.8768, Figure 5B). Consistent with the histological findings, qPCR analysis demonstrated a substantial upregulation of *Iba-1* mRNA expression following *Blautia faecis* intervention without ABX (*t_4_* = 7.740, *p* = 0.0015, Cohen’s d = −6.3200, Figure 5C), alongside trending increases in *Tmem119* and the pro-inflammatory cytokine *IL-1β* (Figures 5D,E). These data indicate that *Blautia faecis* colonization promotes neuroinflammation, aligning with its detrimental impact on cognitive function.

**Figure 6:**
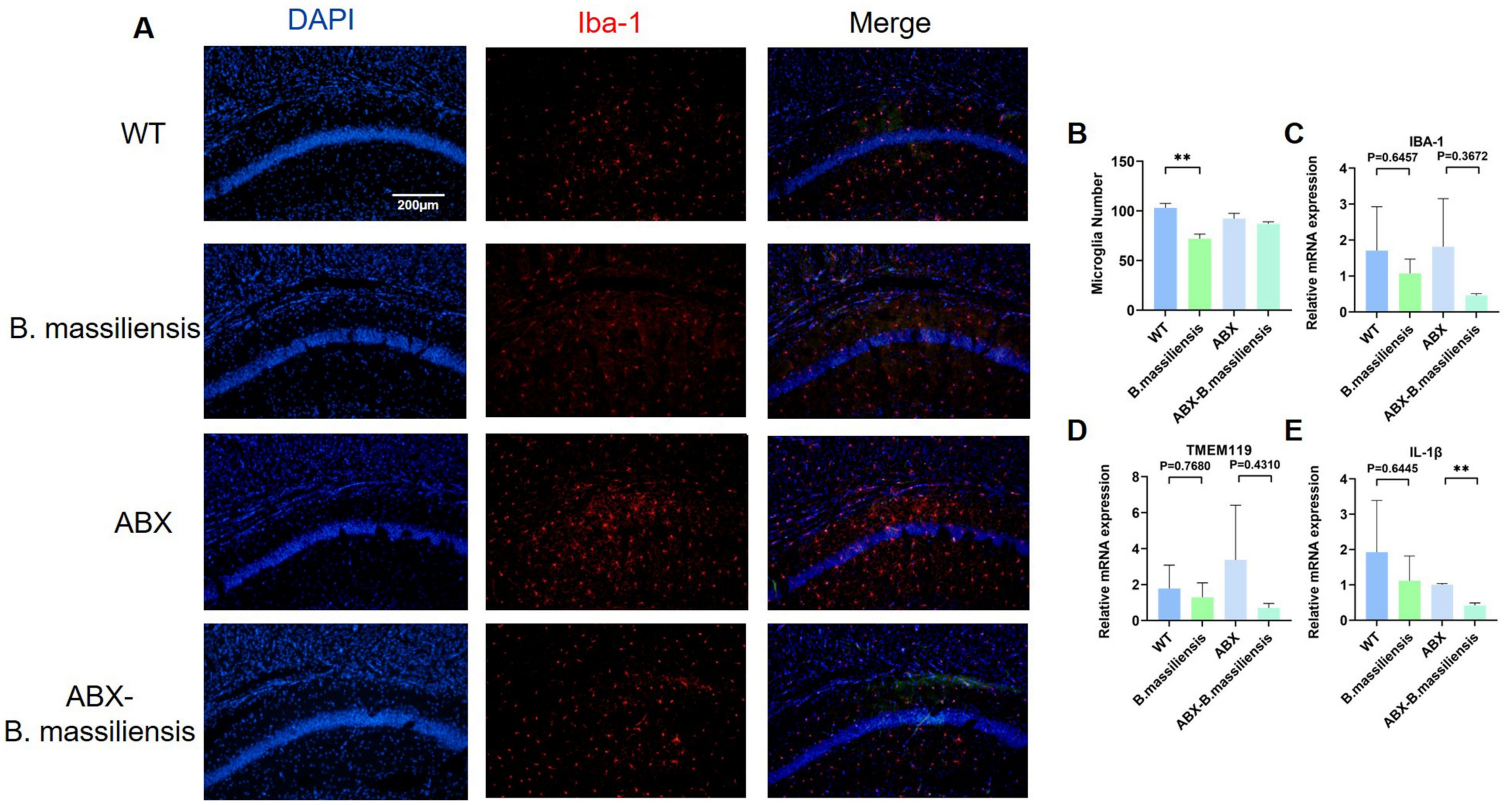
*Blautia massiliensis* reduces the number of microglia and pro-inflammatory factors in the hippocampus. **(A)** Representative immunofluorescence images showing DAPI (blue) and Iba-1 (red) staining in the hippocampus following *B. massiliensis* intervention, Scale bar, 200 μm. **(B)** Quantifications of microglia counts and relative mRNA expression levels of *Iba-1* **(C)**, *Tmem119* **(D)**, and *IL-1β* **(E)** as determined by qPCR normalized to *GAPDH*, with the corresponding vehicle control group set as the calibrator (relative value = 1.0). Specific *p* values for mRNA expression include: *Iba-1* (*p* = 0.6457 for *B. massiliensis* vs. WT; *p* = 0.3672 for ABX-*B. massiliensis* vs. ABX), *Tmem119* (*p* = 0.7680 for *B. massiliensis* vs. WT; *p* = 0.4310 for ABX-*B. massiliensis* vs. ABX), and *IL-1β* (*p* = 0.6445 for *B. massiliensis* vs. WT; *p* < 0.01 for ABX-*B. massiliensis* vs. ABX). Data represent Mean ± SEM (*n* = 3 per group) with statistical significance determined by Student’s t-test, **p < 0.01.

In stark contrast, *Blautia massiliensis* intervention exerted a potent anti-inflammatory effect. In mice without ABX treatment, *Blautia massiliensis* significantly suppressed microglial activation (Figure 6A), evidenced by a marked reduction in Iba-1 positive cell counts compared to the vehicle controls (*t_4_* = 4.709, *p* = 0.0092, Cohen’s d = 3.8451, Figure 6B). Although the reductions in microglia numbers and *Iba-1*/*Tmem119* gene expression under the ABX condition did not reach statistical significance (Figures 6C,D), we conservatively treat this null result as inconclusive due to potential Type II errors in our small cohort. Nevertheless, *Blautia massiliensis* intervention robustly and significantly downregulated the expression of the pro-inflammatory cytokine *IL-1β* in the ABX model (*t_4_* = 7.866, *p* = 0.0014, Cohen’s d = 6.4225, Figure 6E). Collectively, these findings suggest that the cognitive enhancements driven by *Blautia massiliensis*, and the deficits caused by *Blautia faecis*, are closely associated with their differential capacities to modulate the hippocampal neuroinflammatory microenvironment.

### 3.4 *Blautia massiliensis* produces substantially greater levels of acetate than *Blautia faecis*

**Figure 7:**
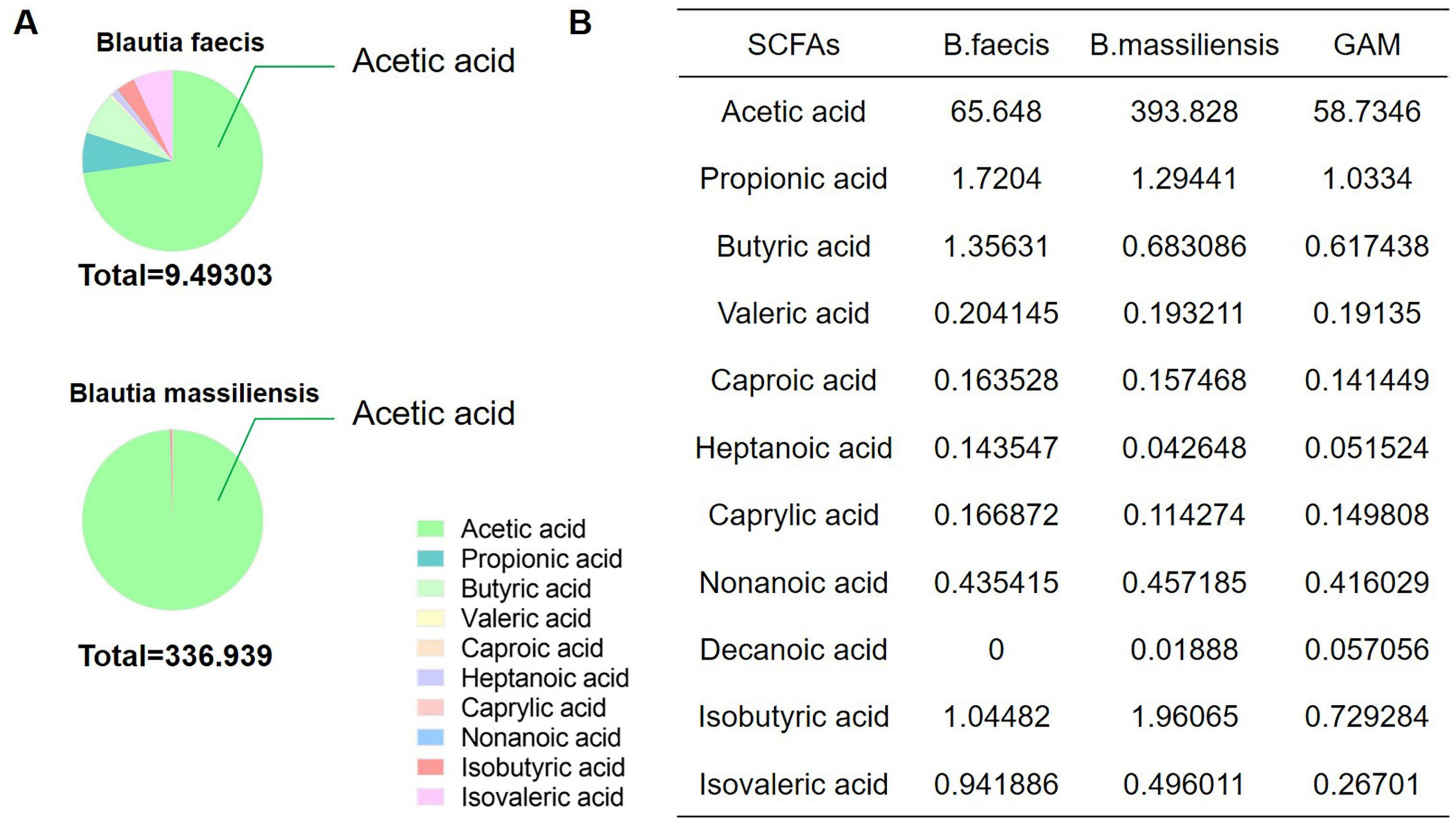
*Blautia massiliensis* produces significantly higher acetate levels than *Blautia faecis*. **(A)** Pie charts illustrating the relative composition and total net concentration (μg/mL) of SCFAs produced by *B. faecis* and *B. massiliensis*, *n* = 1 per group. **(B)** Quantitative table detailing the raw absolute concentrations of 11 specific SCFAs, including acetic, propionic, butyric, valeric, caproic, heptanoic, caprylic, nonanoic, decanoic, isobutyric, and isovaleric acids, detected in the samples via GC–MS using an Agilent 7890B-5977B system equipped with an HP-FFAP capillary column.

To investigate the potential mechanism underlying the opposing effects of *Blautia faecis* on cognitive impairment and *Blautia massiliensis* on cognitive improvement, we quantitatively profiled the short-chain fatty acids (SCFAs) secreted by *Blautia faecis* and *Blautia massiliensis in vitro* using gas chromatography–mass spectrometry (GC–MS). A total of 11 SCFAs were detected. Figure 7B details the raw absolute concentrations of 11 targeted SCFAs detected in the bacterial culture supernatants and the blank General Anaerobic Medium (GAM). To accurately evaluate the actual production capacity of the strains, we calculated their net SCFA secretion by subtracting the GAM background levels.

Notably, acetate levels exhibited a marked difference between the two strains, with *Blautia massiliensis* (393.828 ug/ml) producing substantially higher amounts of acetate than *Blautia faecis* (65.648 ug/ml) as visualized in the pie charts (Figure 7A). Previous studies have demonstrated that acetate can alleviate cognitive impairment by modulating neuroinflammation and enhancing synaptic plasticity (25, 26). Consistent with the observed cognitive dichotomy in our study, These findings suggest that differential acetate production may be linked to the divergent cognitive effects observed for *Blautia faecis* and *Blautia massiliensis*.

## 4 Discussion

**Figure 8:**
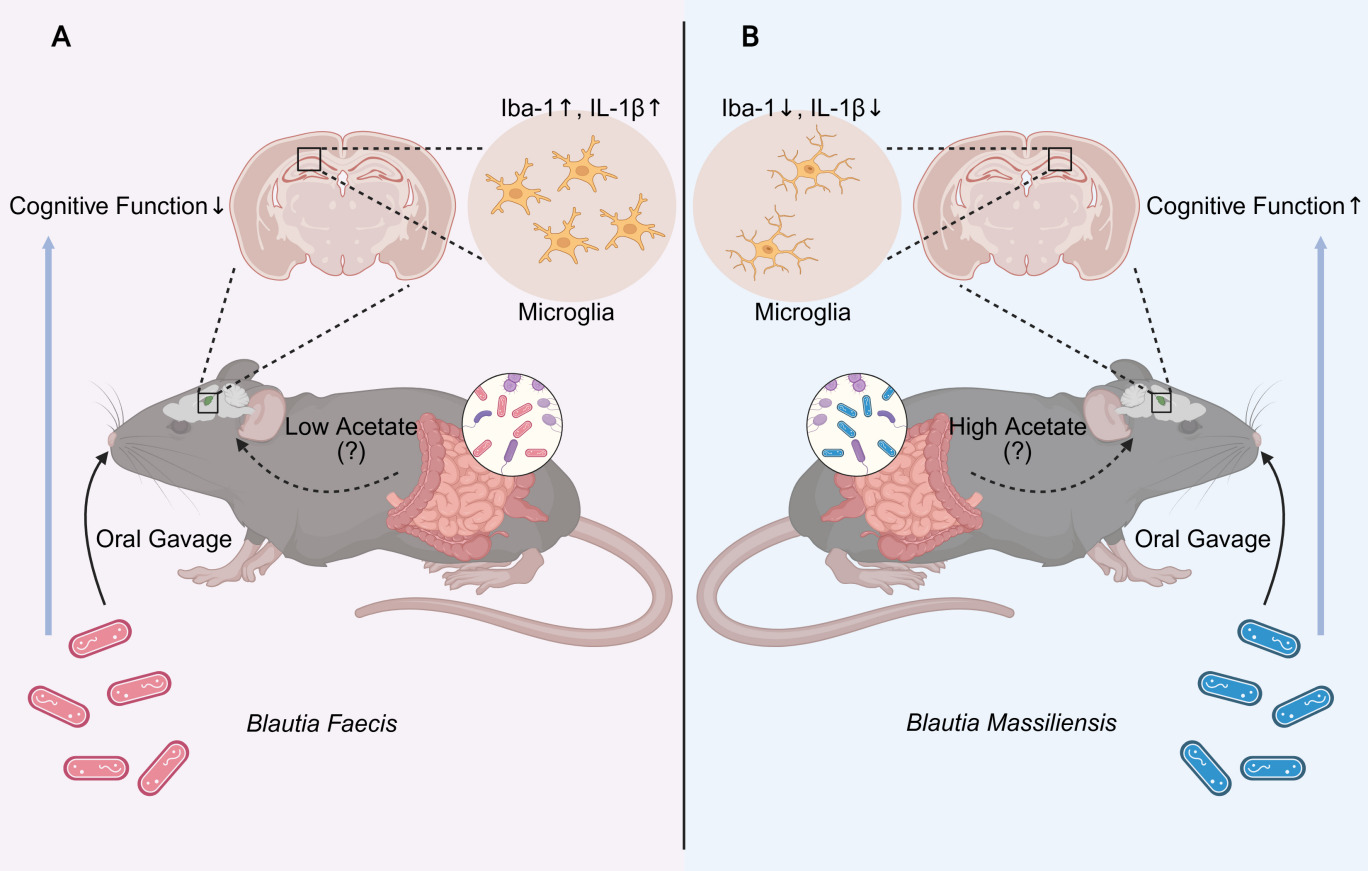
Schematic summary of the study findings. This figure summarizes the key results of the present study, illustrating the potential and opposing effects of *Blautia faecis* and *Blautia massiliensis* on the gut-brain axis. **(A)** *Blautia faecis* exhibits low *in vitro* acetate production and is associated with an increased number of Iba-1^+^ microglia and upregulated pro-inflammatory IL-1β in the hippocampus, which may contribute to impaired cognitive performance. **(B)** Conversely, *Blautia massiliensis* produces substantially greater levels of acetate *in vitro* and is linked to suppressed hippocampal microglial activation (reduced Iba-1 and IL-1β), potentially promoting cognitive improvement. The dashed lines and question marks (?) denote that the direct *in vivo* link between acetate production and neuroimmune modulation remains a hypothesized pathway that warrants further experimental validation.

*Blautia faecis* and *Blautia massiliensis* exert opposing effects on cognitive function, which are potentially linked to thei**r** differential regulation of neuroinflammation and acetate metabolism. *Blautia massiliensis* improves cognition by increasing acetate production and suppressing neuroinflammation, whereas *Blautia faecis* impairs cognition via low acetate output and pro-inflammatory effects. These findings provide a preliminary theoretical basis for understanding how specific *Blautia* strains might modulate cognitive-related neural pathways. Collectively, our data are consistent with the hypothesis that differential acetate production by these specific *Blautia* strains may contribute to their opposing regulatory effects on hippocampal neuroinflammation and cognitive function (Figure 8).

Recent research demonstrates that alterations in *Blautia* abundance and species composition are significantly correlated with cognitive dysfunction across diverse populations. Cross-sectional studies in preclinical Alzheimer’s disease populations, including those with subjective cognitive decline and mild cognitive impairment, consistently report reduced *Blautia* levels compared to cognitively healthy controls (27, 28). The abundance of *Blautia* was positively correlated with standardized cognitive scores, such as the Montreal Cognitive Assessment and Mini-Mental State Examination (29). *Blautia stercoris* MRx0006 attenuates social deficits while also decreasing repetitive and anxiety-like behavior in the autism-relevant, genetic mouse model (12). Given that *Blautia* encompasses multiple species with potentially diverse functional properties, species-level resolution is critical for understanding their precise roles in host health. Accordingly, we examined the cognitive effects of *Blautia massiliensis* and *Blautia faecis* at the species level, offering novel insights into how different members of this genus modulate cognitive function. We observed a behavioral discrepancy between the two paradigms: while *Blautia massiliensis* significantly improved spatial working memory in the Y-maze, it did not yield a statistically significant enhancement in the NOR discrimination index. This divergence likely reflects the distinct cognitive constructs assessed by each test. The Y-maze is highly dependent on the hippocampal CA1 subfield (30), whereas the 30-min NOR paradigm relies more heavily on the perirhinal cortex (31, 32). Additionally, as discussed in the limitations, this non-significant NOR outcome must be interpreted strictly as inconclusive rather than as evidence of absence, as it likely stems from potential Type II errors resulting from our ethically restricted, modest sample size.

*Blautia* appears to modulate cognitive function primarily through its metabolic products, particularly short-chain fatty acids (SCFAs) like acetate. Acetic acid exerts significant anti-inflammatory effects in the regulation of neuroinflammation (33). Studies indicate that higher *Blautia* abundance correlates with increased anti-inflammatory interleukin-10 levels, potentially mitigating excessive neuroinflammatory responses that damage cognitive circuits (28). Acetate produced by the gut microbiota alleviates cognitive impairment through inhibiting of neuroinflammation, modulating of microglial functional status and subsequent reducing of chronic neuroinflammation-induced neuronal damage (33–35). Gut-derived acetate readily crosses the blood–brain barrier, acting as both an energy substrate and a signaling molecule within the central nervous system (36). In the brain microenvironment, microbiota-derived SCFAs are essential for the homeostatic maturation and functional regulation of microglia, a process primarily mediated through specific host receptors such as GPR43 (FFAR2) (37). Previous studies have linked the SCFA-dependent modulation of microglial states to the suppression of neuroinflammation and the promotion of cognitive resilience (38). Our findings align with this growing body of evidence, highlighting how microbial metabolites regulate microglial activation to maintain neuroimmune balance (39).

Notably, our findings are currently limited to the observation that *Blautia massiliensis* decreases microglial numbers and inhibits pro-inflammatory cytokine expression, while *Blautia faecis* shows the opposite effects, with acetate production being markedly higher in *Blautia massiliensis* than in *Blautia faecis*. The complex expression pattern of *Tmem119* observed in our study warrants further discussion. Although classically defined as a homeostatic marker that decreases upon activation, *Tmem119* mRNA exhibited a trending increase alongside *Iba-1* following *Blautia faecis* administration. Given that our transcriptional analysis utilized bulk hippocampal tissue, this trend likely reflects the overall expansion of the microglial population rather than an upregulation at the single-cell level. This pattern also highlights the presence of microglial heterogeneity and transitional activation states. Unlike acute brain injuries that typically induce a uniform loss of homeostatic markers, the chronic, low-grade dysbiosis modeled here may drive microglia into intermediate states, where they initiate pro-inflammatory signaling without fully shedding their homeostatic molecular signatures. This aligns with recent paradigm shifts in neuroimmunology, which emphasize that microglia exist in highly dynamic, complex functional phenotypes beyond a simple binary classification of ‘resting’ versus ‘activated’. Nevertheless, because we did not directly quantify *in vivo* SCFA concentrations in relevant biological matrices (such as feces or brain tissue), whether acetate-driven neuroimmune modulation accounts for the opposing cognitive effects of *Blautia massiliensis* and *Blautia faecis* has not been experimentally validated. Hence, further research employing integrated strategies is needed to dissect the mechanistic basis underlying the differential roles of *Blautia massiliensis* and *Blautia faecis* in cognitive regulation.

Interestingly, we observed that both *Blautia massiliensis* and *Blautia faecis* exhibited higher colonization efficiency in naïve mice compared to antibiotic-pretreated mice, suggesting a complex host-microbiota interaction underlying this phenomenon. Although antibiotic pretreatment is intended to clear the indigenous microbiota to create a niche for donor strains, its disruption of the intestinal microenvironment may paradoxically impair donor strain engraftment (40, 41). By contrast, untreated C57BL/6J mice harbor a stable and homeostatic microbial ecosystem (42). Consistently, previous research comparing antibiotic pretreatment durations for fecal microbiota transplantation reported that a 3-week regimen did not enhance donor strain engraftment relative to a 3-day regimen, and instead delayed the recovery of the recipient’s gut microbiota (43). Beyond reduced colonization efficiency, the absence of cognitive and neuroinflammatory effects in the ABX-pretreated model suggests several alternative mechanisms. First, the functional efficacy of *Blautia* may depend on synergistic interactions or cross-feeding networks with indigenous microbes that are eradicated by broad-spectrum antibiotics (44). Second, ABX treatment severely disrupts the intestinal epithelial niche, potentially altering the local mucosal microenvironment including pH, oxygen gradients, and mucin layer integrity. Such changes may prevent these strictly anaerobic strains from sustaining the metabolic activities necessary for acetate production (45). Finally, the prolonged antibiotic cocktail itself exerts direct effects on host neuroimmunity and cognition. This systemic depletion of commensal-derived metabolites may create a baseline physiological disruption that masks the subtle modulatory effects of an individual bacterial strain (46).

## 5 Limitations and future directions

Despite the insights gained from this study, several limitations warrant acknowledgment and guide future research. First, no significant difference in the discrimination index was observed in the novel object recognition test following *Blautia massiliensis* administration in mice without antibiotic pretreatment. The small sample size (*n* = 6 per group) and associated inter-individual variability may have limited the statistical power to detect a significant effect. A retrospective power analysis indicates that reliably detecting a conventional medium effect size (*f* = 0.25 in our 2 × 2 factorial design) would require a significantly larger cohort of 128 animals (*n* = 32 per group). Because our exploratory study was constrained by ethical imperatives to minimize animal use (*n* = 6 per group), it is fundamentally underpowered to detect subtle or medium biological variations. Consequently, any non-significant outcomes in our behavioral paradigms must be interpreted with caution and are conservatively regarded as inconclusive due to the high risk of Type II errors, rather than as definitive evidence of the absence of an effect. Future investigations with expanded sample cohorts are necessary to consolidate these preliminary observations. Second, while we used wild-type C57BL/6 mice as a model to investigate the effects of bacterial interventions on cognitive function, this model may not fully recapitulate the pathological context of cognitive impairment. The 5xFAD transgenic mouse model which harbors five familial AD mutations and exhibits robust amyloid-beta (Aβ) deposition, neuroinflammation, and progressive cognitive decline, would provide a more clinically relevant framework to evaluate gut microbiota-mediated cognitive modulation in a disease context. In subsequent studies, we will utilize 5xFAD mice to investigate the effects of these *Blautia massiliensis* and *Blautia faecis* on cognitive function. Third, our experimental design exclusively utilized male mice to minimize hormonal variance. Given the established sex-dependent differences in gut microbiota composition, immune function, and cognitive performance, our current findings cannot be broadly generalized to both sexes. Future studies incorporating both male and female cohorts are essential to comprehensively evaluate potential sex-dimorphic effects of these *Blautia* strains on the gut-brain axis (17, 18). Fourth, the use of a 7-day broad-spectrum antibiotic cocktail to deplete commensal microbiota, while effective for engraftment, was not verified for depletion efficacy via culture or 16S sequencing. Furthermore, such antibiotic treatment can induce intestinal mucosal injury such as villous atrophy or increased permeability, which might independently influence cognitive function and neuroinflammation. Although we included an ABX-only group to control for these background effects, future studies should employ extended antibiotic pretreatment protocols or germ-free models, combined with comprehensive microbiome profiling, to validate depletion efficiency and minimize potential confounding physiological impacts. A significant limitation of our study is the lack of post-intervention whole-microbiome profiling (16S rRNA sequencing). While our PCR-based validation confirmed the successful engraftment of *Blautia faecis* and *Blautia massiliensis*, we cannot definitively exclude the possibility that the administered strains induced secondary shifts in the indigenous microbial community, which might contribute to the observed neurocognitive and immune phenotypes. Future studies integrating 16S rRNA or metagenomic sequencing are required to decouple the direct effects of the administered strains from community-wide microbial restructuring. It is also worth noting that while our experimental design utilized WT and *Blautia faecis*-treated mice to induce and evaluate cognitive shifts, the inclusion of a pharmacological positive control for cognitive impairment such as scopolamine-treated mice would have further validated the sensitivity of our behavioral paradigms. Nevertheless, the significant cognitive deficits observed in the *Blautia faecis* groups, when compared to the WT control, provide empirical evidence that our behavioral paradigms were sufficiently sensitive to detect and distinguish impaired cognitive performance. Furthermore, our analysis of Iba-1 + microglia was restricted to the hippocampal CA1 subfield. While the CA1 region is critically involved in spatial working memory (assessed by the Y-maze) and exhibits profound selective vulnerability to metabolic and inflammatory stress, we acknowledge that this focus does not capture the full neuroinflammatory profile of the entire hippocampus (e.g., dentate gyrus and CA3) or related cognitive regions like the prefrontal cortex and perirhinal cortex (30, 47). Future studies should employ a broader regional analysis to provide a more holistic mapping of *Blautia*-mediated neuroimmune responses across the cortico-hippocampal network (43). Besides, the structural integrity of the intestinal and blood–brain barriers was not evaluated in this study; future research should incorporate barrier-related markers to further clarify the transport mechanisms of microbial metabolites.

Finally, we must strictly emphasize that this study represents an early preclinical proof-of-concept. The observation of cognitive modulation in a small cohort of healthy wild-type mice cannot be directly extrapolated to human therapeutics. A substantial gap remains between our current findings and clinical application. Extensive future research including validation in specific disease-relevant models, rigorous safety profiling, comprehensive dose optimization, and eventual human clinical trials is absolutely required before considering *Blautia massiliensis* as a viable candidate for probiotic intervention in related cognitive disorders.

## Data Availability

The original contributions presented in the study are included in the article/Supplementary material, further inquiries can be directed to the corresponding authors.
